# Acid Stripping of Surface IgE Antibodies Bound to FcεRI Is Unsuitable for the Functional Assays That Require Long-Term Culture of Basophils and Entire Removal of Surface IgE

**DOI:** 10.3390/ijms21020510

**Published:** 2020-01-13

**Authors:** Caroline Galeotti, Anupama Karnam, Mrinmoy Das, Srini V. Kaveri, Jagadeesh Bayry

**Affiliations:** 1 Institut National de la Santé et de la Recherche Médicale; Centre de Recherche des Cordeliers, Equipe-Immunopathologie et Immunointervention Thérapeutique, Sorbonne Université, F-75006 Paris, France; caroline.galeotti@gmail.com (C.G.); suma.anupama@gmail.com (A.K.); mdasmicro@gmail.com (M.D.); srini.kaveri@crc.jussieu.fr (S.V.K.); 2 Service de Rhumatologie Pédiatrique, Centre de Référence des Maladies Auto-Inflammatoires Rares et des Amyloses, CHU de Bicêtre, le Kremlin Bicêtre, F-94270 Paris, France; 3 Université Paris Descartes, Sorbonne Paris Cité, F-75006 Paris, France

**Keywords:** IgE, basophil, allergy, atopy, inflammation, human, IL-3, activation, lactic acid, acetic acid, stripping, CD123, viability

## Abstract

Basophils are rare granulocytes and dysregulated functions of these cells are associated with several atopic and non-atopic allergic diseases of skin, respiratory system and gastrointestinal tract. Both cytokines and immunoglobulin E (IgE) are implicated in mediating the basophil activation and pathogenesis of these disorders. Several reports have shown that healthy individuals, and patients with allergic disorders display IgG autoantibodies to IgE and hence functional characterization of these anti-IgE IgG autoantibodies is critical. In general, anti-IgE IgG autoantibodies modulate basophil activation irrespective of allergen specificity by interacting with constant domains of IgE. Therefore, an ideal solution to prove the functions of such anti-IgE IgG autoantibodies would be to completely eliminate type I high affinity immunoglobulin E receptor (FcɛRI)-bound IgE from the surface of basophils and to demonstrate in an unequivocal manner the role of anti-IgE IgG autoantibodies. In line with previous reports, our data show that FcɛRI on peripheral blood basophils are almost saturated with IgE. Further, acetic acid buffer (pH 4) efficiently removes these FcɛRI-bound IgE. Although immediately following acetic acid-elution of IgE had no repercussion on the viability of basophils, following 24 h culture with interleukin-3 (IL-3), the viability and yield of basophils were drastically reduced in acid-treated cells and had repercussion on the induction of activation markers. Lactic acid treatment on the other hand though had no adverse effects on the viability of basophils and IL-3-induced activation, it removed only a small fraction of the cell surface bound IgE. Thus, our results show that acid buffers could be used for the elution of FcɛRI-bound IgE on the basophil surface for the biochemical characterization of IgE antibodies or for the immediate use of basophils to determine their sensitivity to undergo degranulation by specific allergens. However, these methods are not utile for the functional assays of basophils that require longer duration of culture and entire removal of surface IgE to validate the role of anti-IgE IgG autoantibodies that interact with FcɛRI-bound IgE irrespective of allergen specificity.

## 1. Introduction

Basophils are rare granulocytes and have various functions in the orientation of immune responses by regulating the macrophage polarization, supporting the CD4^+^ T helper 2 (Th2) and B cell differentiation, immunoglobulin (Ig) class-switch and memory responses. Basophils express diverse receptors such as type I high affinity immunoglobulin E receptor (FcεRI) that binds to IgE, toll-like receptors that recognize pathogen-associated molecular patterns (PAMPs), and cytokine receptors including the receptors for interleukin (IL)-3, IL-33 and thymic stromal lymphopoietin (TSLP) to sense activation signals derived from the varied sources [1,2]. Activated basophils release several inflammatory mediators including various cytokines, histamine and lipid mediators like leukotriene. Dysregulated functions of basophils are associated with several atopic (characterized by high allergen-specific IgE) and non-atopic allergic (non IgE-mediated) diseases of skin, respiratory system and gastrointestinal tract (allergic rhinitis, atopic dermatitis, IgE-mediated chronic allergic inflammation) and autoimmune/inflammatory pathologies (bullous pemphigoid, eosinophilic oesophagitis, lupus nephritis, allograft rejection) [1,2,3,4,5,6,7,8]. Both cytokines and IgE are implicated in mediating the basophil activation and pathogenesis of these disorders.

IgE is the least represented immunoglobulin class. With a half-life of 1.5 days, IgE accounts for nearly 0.05% of total immunoglobulins in the body [9]. Despite its low representation, IgE has several important functions in pathophysiologies. IgE in conjunction with basophils are critical mediators of protective immune response to parasites. On the other hand, IgE antibodies are well known for their role in eliciting allergic reactions [9]. Cross-linking of FcεRI-bound IgE by allergens provide potent signal for the degranulation of basophils and mast cells leading to the release of histamine, lipid mediators such as leukotriene C4, and cytokines like IL-4 [1,2]. IgE antibodies to dsDNA and basophils are also implicated in the pathogenesis of lupus nephritis [10,11]. Therefore, IgE has been explored as one of the therapeutic targets in recent years to treat allergic and inflammatory diseases [12]. Several anti-IgE molecules including disruptive IgE inhibitors are currently being considered for the allergic pathologies [12].

Several reports over the years have shown that patients with allergic disorders display IgG autoantibodies to IgE, which either activate IgE-sensitised basophils (agonistic anti-IgE IgG autoantibodies) or inhibit allergen-induced basophil activation (antagonistic anti-IgE IgG autoantibodies) in part by blocking the binding of specific allergen to IgE [13,14,15]. IgG autoantibodies that react with high-affinity receptor for IgE, i.e., FcεRI are also identified in healthy individuals and atopic diseases [16,17] suggesting that IgG autoantibodies to IgE and FcεRI might co-operate to regulate basophil functions. In line with these reports, our recent report shows that IgG autoantibodies to IgE are present in the healthy individuals [18,19]. By using therapeutic normal IgG or intravenous immunoglobulin (IVIG), obtained from the pooled plasma of thousands of healthy donors [20], we showed that normal IgG induces activation of IL-3-primed human basophils by signalling through FcɛRI-bound IgE [18]. Depletion of such anti-IgE IgG autoantibodies compromised the ability of IVIG to induce activation of IL-3-primed basophils.

Functional characterization of anti-IgE IgG antibodies-induced FcɛRI-IgE signalling events in the context of basophil functions is critical for dissecting the role of these anti-IgE IgG autoantibodies in pathophysiologies. Various lines of evidences now suggest that anti-IgE IgG autoantibodies modulate human basophil activation and function not by interacting with variable region of IgE rather by recognizing constant domains of heavy chains of IgE, in particular Cε1, Cε2 or Cε4 domains [14,19,21]. Therefore, in view of the fact that anti-IgE IgG autoantibodies modulate basophil activation irrespective of allergen specificity, an ideal solution to functionally characterize such anti-IgE IgG autoantibodies would be to completely eliminate FcɛRI-bound IgE from the surface of basophils and to demonstrate in an unequivocal manner the role of anti-IgE IgG autoantibodies.

Previous studies have attempted to elute IgE from the surface of basophils by brief treatment of cells with either 0.01 M lactic acid-containing buffer (pH 3.9) or acetic acid buffer (pH 2.5 to 4) [22,23,24,25,26,27,28]. Although, post-treated basophils were immediately used for various purposes like to determine the sensitivity of basophils to undergo degranulation by specific allergens or for imaging purposes, it is not known whether such acid buffer-treated basophils remain viable and hence could be used in functional assays that require basophil culture for longer periods. Therefore, we investigated the efficacy of IgE stripping by these two approaches and the viability of basophils over 24 h.

## 2. Results

### 2.1. FcɛRI on Basophils Are Almost Saturated with IgE

**Figure 1:**
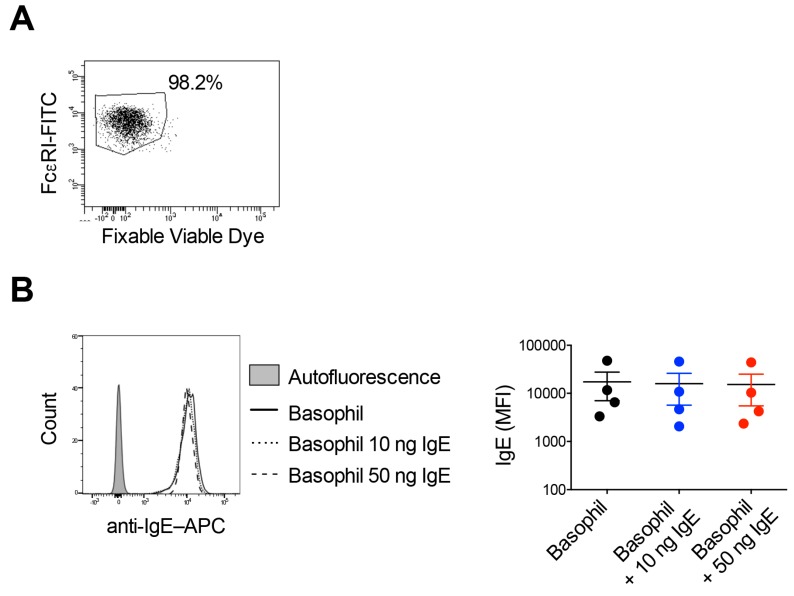
FcɛRI on basophils are almost saturated with IgE. (**A**) The viability of isolated basophils as analyzed by fixable viable dye staining. (**B**) Peripheral blood basophils were incubated with extrinsic amounts of IgE (10 ng or 50 ng/million cells) for 30 min and followed by surface staining for IgE on the basophils. The intensity of IgE binding on the basophils was analyzed by flow cytometry. Representative histogram overlays (left panel) and the intensity of IgE on the surface of basophils as represented by MFI values (Median fluorescence intensity, mean ± SD, *n* = 4 donors) (right panel).

The viability of basophils following isolation from the buffy bags of healthy donors was in the range of 98–99% as analyzed by fixable viable dye (FVD) staining (Figure 1A). Previous study has reported that ~97% (on median basis) of high-affinity FcεRI on the basophils are occupied with IgE [29]. Other reports have also suggested that the expression of FcɛRI on the basophils is directly related to IgE levels [22,30]. As shown in the Figure 1B, basophils from the healthy donors displayed high levels of surface-bound IgE. Further, to explore if FcɛRI on basophils are almost saturated with IgE, we incubated the basophils with exogenous recombinant human IgE for 30 min and followed by surface staining for IgE on the basophils by flow cytometry. However, there were no major differences in the intensity of IgE on the basophils if they were incubated with additional IgE (10 or 50 ng) or not (Figure 1B), thus indicating that all FcɛRI on basophils are already saturated/occupied by IgE.

### 2.2. Stripping of Surface IgE Antibodies Bound to FcεRI of Basophils by Acetic Acid Buffer (pH 4)

**Figure 2:**
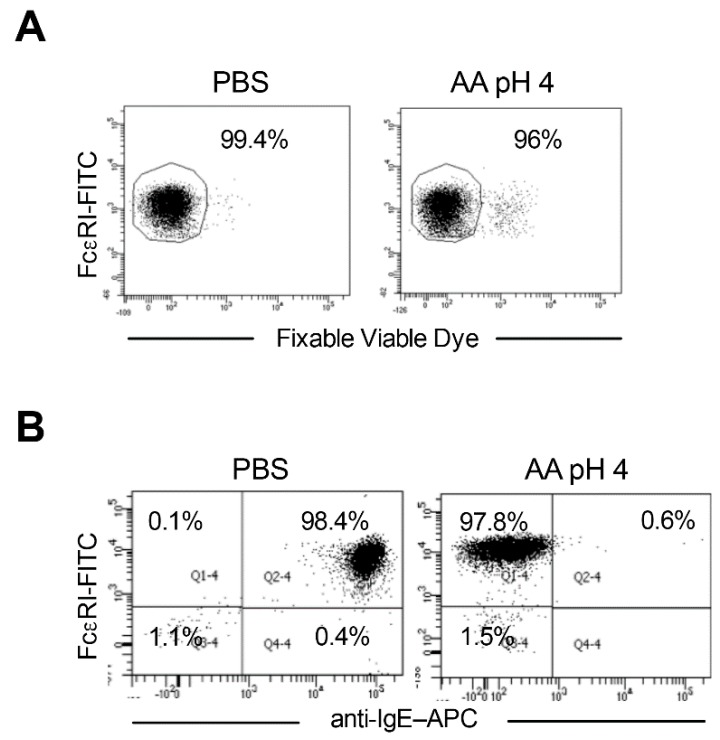
Stripping of surface IgE antibodies bound to FcεRI of basophils by acetic acid buffer (pH 4). Basophils were incubated on ice for 5 min either with phosphate buffered saline (PBS) or ice-cold acetic acid buffer pH 4 (0.05 M acetate, 0.085 M NaCl, 0.01 M EDTA and 0.03% human serum albumin) (AA pH 4). Cells were then washed and proceeded with phenotype analyses by flow cytometry. (**A**) The viability of cells immediately following AA pH 4 treatment as analyzed by fixable viable dye staining. (**B**) Efficacy of stripping of basophil surface-bound IgE by AA pH 4 (right panel) as analyzed by surface staining of IgE and analyses by flow cytometry. Representative data from four donors are presented.

We analyzed the viability of basophils immediately following acetic acid buffer (pH 4) treatment by staining with FVD. We did not observe major changes in the viability of acid-treated basophils compared to phosphate-buffered saline (PBS)-treated cells (Figure 2A).

We then assessed the efficacy of stripping of basophil surface-bound IgE. Interestingly, treatment of cells with acetic acid buffer (pH 4) led to almost complete stripping of IgE from the basophil surface (Figure 2B). Over 99% of acid-treated basophils became negative for the surface IgE advocating that acetic acid buffer (pH 4) has effectively eluted FcεRI-bound IgE antibodies from the peripheral blood basophils.

### 2.3. Response of the IgE Stripped Human Peripheral Blood Basophils to IL-3 Stimulation

IL-3 plays an important role in the biology of basophils [31,32,33,34,35]. In addition to providing survival signals, IL-3 is the most potent inducer of activation of human basophils among all other cytokines. IL-3 priming is also a prerequisite for the IgE-mediated degranulation and for the activation induced by circulating normal IgG [18,31]. Therefore, the important question was whether IgE stripped basophils could be used for the functional assays, in particular to employ them as ‘IgE-deficient basophils’ to authenticate the ability of anti-IgE IgG autoantibodies to induce basophil activation.

**Figure 3:**
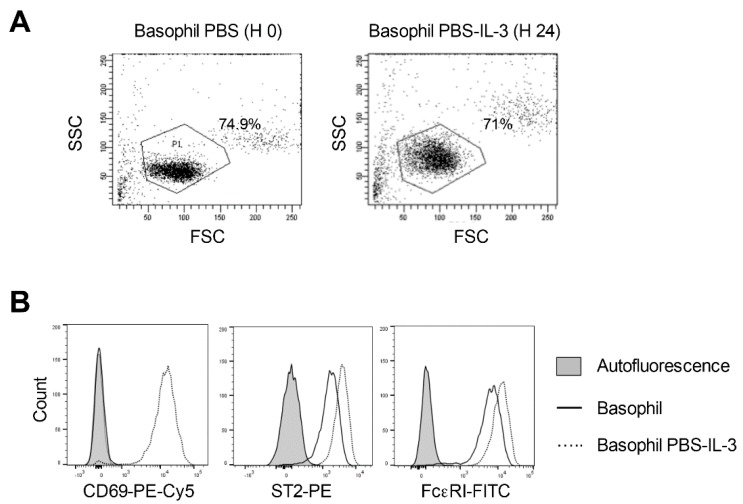
Response of the phosphate buffered saline (PBS)-treated human peripheral blood basophils to IL-3 stimulation. Basophils were incubated on ice for 5 min with PBS. Cells were then washed and cultured in serum-free X-VIVO 15 medium (0.1 × 10^6^ cells/well per 200 µL) in 96-well U-bottomed plate along with IL-3 (100 ng/0.5 million cells) for 24 h. (**A**) The forward and side scatter plot of the basophils immediately following PBS treatment and following 24-h culture in the presence of IL-3. (**B**) Histogram overlays showing the expression of basophil activation markers CD69, ST2 and FcεRI in PBS-treated basophils cultured for 24 h in IL-3. Representative data from four donors are presented.

Therefore, as a first step, we cultured PBS or acetic acid buffer (pH 4)-treated basophils in IL-3 for 24 h. Analyses of basophils by flow cytometry revealed that PBS-treated basophils remain highly viable after 24 h culture and we observed only minor changes in the cell yield (Figure 3A). In addition, IL-3 induced or enhanced the expression of various activation markers on basophils such as Cluster of Differentiation 69 (CD69), Suppression of Tumorigenicity 2 (ST2) and FcεRI (Figure 3B).

**Figure 4:**
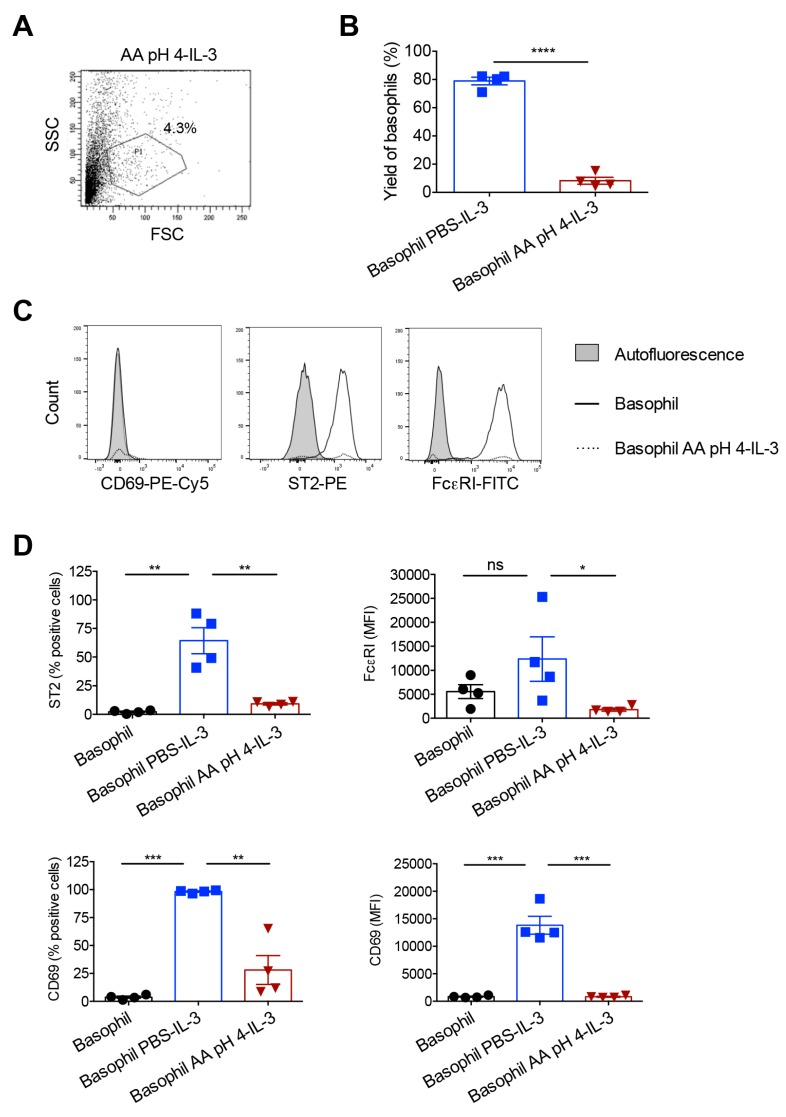
Response of the acetic acid buffer (pH 4)-treated human basophils to IL-3 stimulation. Basophils were incubated on ice for 5 min either with phosphate buffered saline (PBS) or ice-cold acetic acid buffer pH 4 (AA pH 4). Cells were then washed and cultured in serum-free X-VIVO 15 medium (0.1 × 10^6^ cells/well per 200 µL) in 96-well U-bottomed plate along with IL-3 (100 ng/0.5 million cells) for 24 h. (**A**) The forward and side scatter plot of AA pH 4-treated basophils following 24-h culture in IL-3. (**B**) The yield of basophils (mean ± SD, *n* = 4 donors) after 24 h culture of basophils in IL-3. The values were calculated based on the percentage of cells in the P1 gate of forward and side scatter plot. (**C**) Histogram overlays showing the expression of basophil activation markers CD69, ST2 and FcεRI in AA pH 4-treated cells after 24 h culture in IL-3. Representative data from four donors are presented. (**D**) The expression (mean ± SD, *n* = 4 donors) of various activation markers (% positive cells or median fluorescence intensity, MFI) after 24 h culture of basophils in IL-3. * *p* < 0.05; ** *p* < 0.01; *** *p* < 0.001; **** *p* < 0.0001; ns, not significant; two-sided Students t-test (panel B) or one-way ANOVA with Tukey’s multiple comparison test (panel D).

In contrast, the cell yield after 24 h culture in IL-3 was drastically reduced in acetic acid buffer (pH 4)-treated basophils. Based on the forward and side scatter pattern of cells, the percentage of remaining basophils in P1 gate relative to PBS-treated cells was in the range of 8.2 ± 4.9% (*n* = 4 donors) (Figure 4A,B). This low viability also had repercussion on the expression of IL-3-induced basophil activation markers wherein unlike PBS-treated cells, acetic acid buffer (pH 4)-treated basophils did not show enhanced upregulation of CD69, ST2 and FcεRI (Figure 4C,D). These data thus suggest that acetic acid buffer (pH 4) though effective to strip off IgE from the basophil surface, this method is not convenient for the functional assay of basophils due to its adverse effects on the yield of cells following 24 h culture.

**Figure 5:**
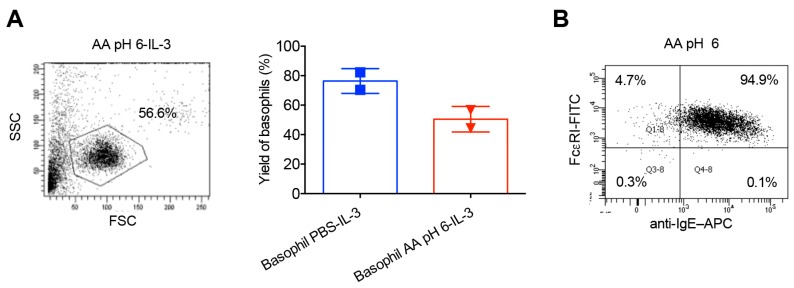
Acetic acid buffer pH 6 improves the viability of basophils but is ineffective to strip off IgE from the basophils. Basophils were incubated on ice for 5 min with ice-cold acetic acid buffer pH 6 (AA pH 6). Cells were then washed and cultured in serum-free X-VIVO 15 medium (0.1 × 10^6^ cells/well per 200 µL) in 96-well U-bottomed plate along with IL-3 (100 ng/0.5 million cells) for 24 h. (**A**) The forward and side scatter plot of AA pH 6-treated basophils following 24-h culture in IL-3 (left panel) and the yield of basophils (mean ± SD, *n* = 2 donors) (right panel). The values were calculated based on the percentage of cells in the P1 gate of forward and side scatter plot. (**B**) Efficacy of stripping of basophil surface-bound IgE by AA pH 6 as analyzed by surface staining of IgE and analyses by flow cytometry.

We then wondered if pH of the acetic acid buffer close to neutral would overcome this problem and hence treated the basophils with acetic acid buffer (pH 6). Although the percentage of remaining basophils in P1 gate following 24 h culture was considerably improved (Figure 5A), acetic acid buffer (pH 6) was infective to strip off surface IgE from the basophils (Figure 5B). Acetic acid buffer (pH 5) was also ineffective to remove IgE from the basophil surface.

### 2.4. Effect of Lactic Acid on the Elution of IgE and the Activation of Basophils

Several studies have performed elution of IgE from the basophil surface by using 0.01 M lactic acid pH 3.9 [23,24,25,26,27,28]. The initial protocol that was aimed at removing entire cell-bound IgE from the basophil surface also damaged the basophils [23,29]. Therefore, we followed the protocol reported by DW MacGlashan Jr, which removes only a small fraction of IgE from the surface [29].

**Figure 6:**
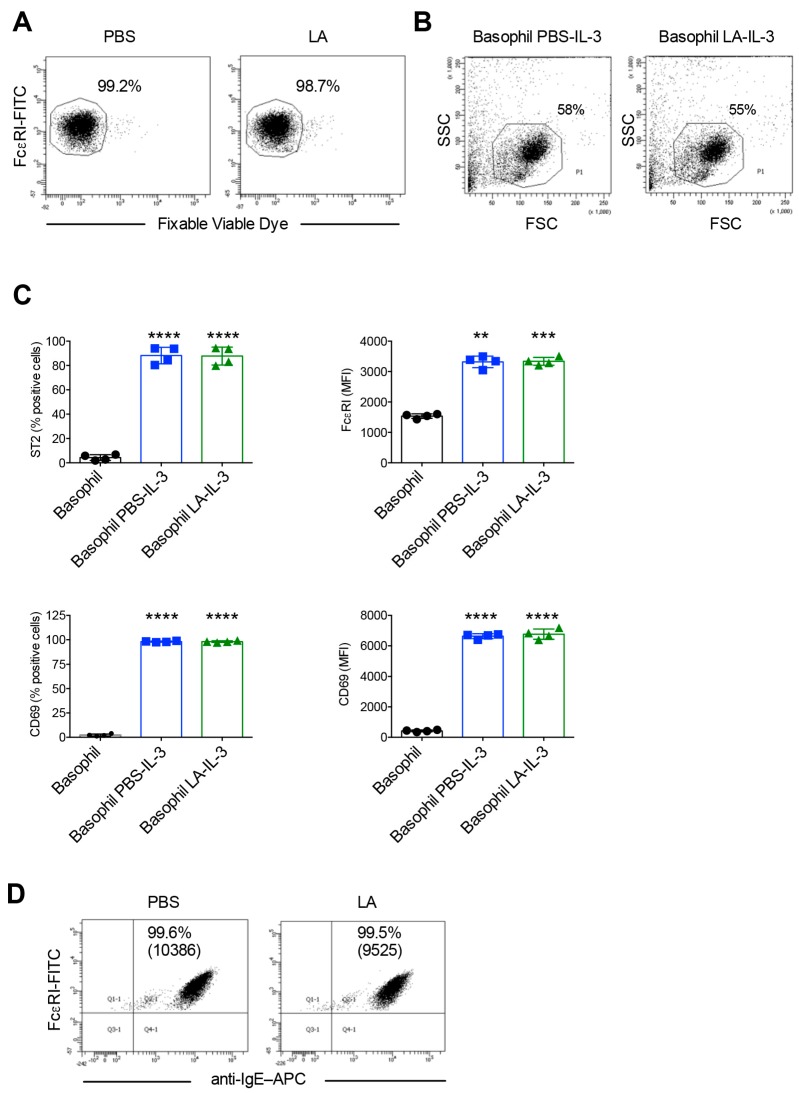
Lactic acid treatment does not affect viability, yield and IL-3 induced activation of basophils, but removes only a small fraction of the cell surface bound IgE. Basophils were incubated on ice either with phosphate buffered saline (PBS) or ice-cold lactic acid buffer pH 3.9 (LA). Cells were then washed and cultured in serum-free X-VIVO 15 medium (0.1 × 10^6^ cells/well per 200 µL) in 96-well U-bottomed plate along with IL-3 (100 ng/0.5 million cells) for 24 h. (**A**) The viability of cells immediately following LA treatment as analyzed by fixable viable dye staining. (**B**) The forward and side scatter plot of LA-treated basophils following 24-h culture in IL-3. (**C**) The expression of basophil activation markers ST2, FcεRI and CD69 in LA-treated cells after 24 h culture in IL-3 (mean ± SD, *n* = 4 experiments from two donors). (**D**) Efficacy of stripping of basophil surface-bound IgE by LA as analyzed by surface staining of IgE and analyses by flow cytometry. The intensity of IgE on the surface of basophils was represented by MFI values (Median fluorescence intensity). ** *p* < 0.01; *** *p* < 0.001; **** *p* < 0.0001; one-way ANOVA with Tukey’s multiple comparison test.

Analyses of viability of basophils immediately following lactic acid treatment confirmed that lactic acid did not affect the viability of basophils (Figure 6A). Furthermore, the yield of basophils (Figure 6B) and the expression of activation markers CD69, ST2 and FcεRI following 24 h culture in the presence of IL-3 (Figure 6C) were similar between PBS-treated and lactic acid-treated basophils. These data thus show that in contract to acetic acid buffer (pH 4), which drastically reduced the basophil yield and response to IL-3 stimulation, lactic acid treatment did not have such adverse effects on the basophils. However, in line with the previous reports, lactic acid eluted only a small fraction of cell surface bound IgE (Figure 6D).

### 2.5. Acetic Acid Buffer (pH 4) and Lactic Acid Differentially Affect the Viability of Basophils Cultured in the Presence of IL-3

**Figure 7:**
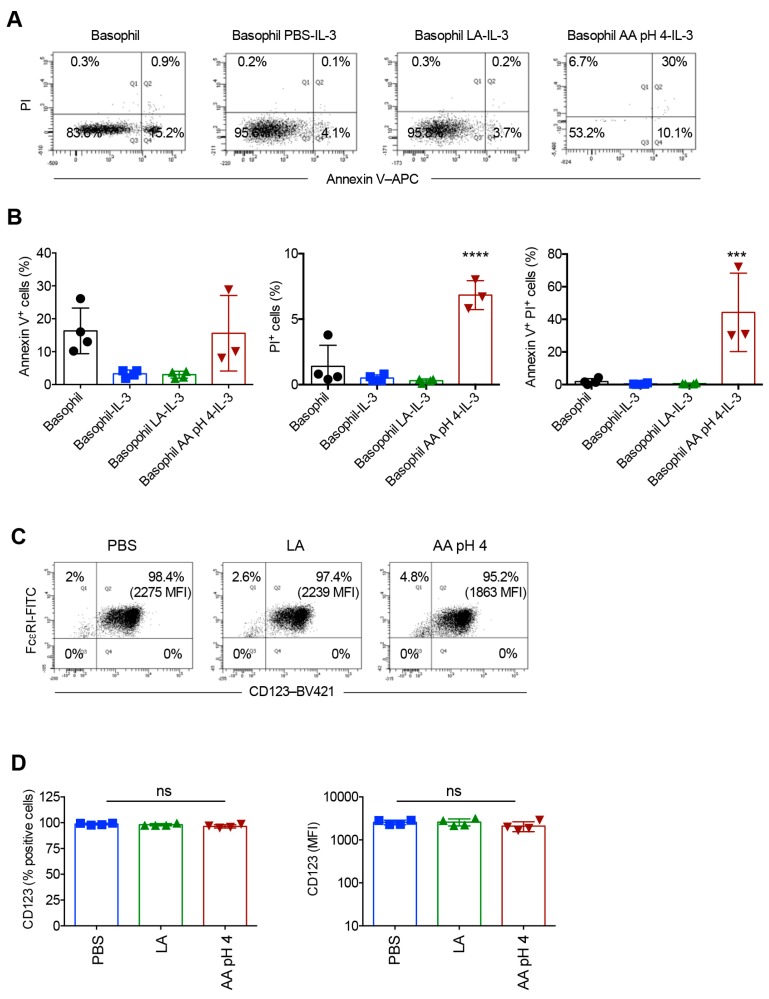
Effect of acetic acid buffer pH 4 (AA pH 4) and lactic acid (LA) treatment on the viability of basophils cultured for 24 h in the presence of IL-3. The viability of basophils was analyzed by Annexin V and PI staining. (**A**) Representative dot plots and (**B**) data (mean ± SD, four experiments from two donors) are presented. In AA pH 4-treatment condition, no viable cells were obtained in one of the experiments. (**C**,**D**) The expression of CD123 (% positive cells and median fluorescence intensity, MFI) on the basophils as analyzed by flow cytometry immediately following treatment of cells with PBS, LA or AA pH 4. (**C**) Representative dot plots and (**D**) data (mean ± SD, four experiments from two donors) are presented. *** *p* < 0.001; **** *p* < 0.0001; ns, not significant; one-way ANOVA with Tukey’s multiple comparison test.

The distinct effects of acetic acid buffer (pH 4) and lactic acid buffer treatments on the yield of basophils cultured for 24 h in the presence of IL-3 prompted us to investigate the viability of cells by Annexin V and propidium iodide (PI) staining. Basophils cultured in the medium alone without supplementation of IL-3 showed high levels of Annexin V positivity (Figure 7A,B). IL-3 on the other hand, rescued the basophils from undergoing apoptosis thus confirming the previous reports (Figure 7A,B) [31]. While lactic acid treatment did not show any adverse effects on the viability of basophils, percentage of cells positive for Annexin V and PI was significantly increased in acetic acid buffer (pH 4)-treated cells (Figure 7A,B). These data thus provide explanation for the drastically reduced cell yield in acetic acid buffer (pH 4)-treated condition. We could get only 20–50 events for the analyses of Annexin V and PI staining in acetic acid buffer (pH 4)-treated basophils.

We wondered whether inability of IL-3 to rescue acetic acid buffer (pH 4)-treated basophils was due to the loss of IL-3 receptor following acid treatment. Therefore, we analyzed CD123 (IL-3Rα) expression on the basophils immediately following treatment of cells with PBS, lactic acid or acetic acid buffer (pH 4). We observed that irrespective of the treatments, basophils retained similar intensity of CD123 expression (Figure 7C,D) thus confirming that low yield of acetic acid buffer (pH 4)-treated basophils cultured for 24 h was due to cell damage caused by the buffer.

## 3. Discussion

Our data show that FcɛRI on peripheral blood basophils are almost saturated with IgE. Hence, the addition of exogenous IgE did not considerably alter the intensity of IgE on the surface. Further, acetic acid buffer (pH 4) efficiently removes these FcɛRI-bound IgE, while buffer with pH 6 was ineffective. Acetic acid buffer (pH 4)-elution of IgE had no immediate repercussion on the viability of basophils but following 24 h culture with IL-3, the yield and viability of basophils was drastically reduced and had repercussion on the induction of activation markers. Lactic acid treatment on the other hand though had no adverse effects on the viability of basophils and IL-3 induced activation, it removed only a small fraction of the cell surface bound IgE. Thus, our results show that although acid treatments could be used for the elution of FcɛRI-bound IgE on the basophil surface for the biochemical characterization of IgE antibodies or for the immediate use of basophils to determine their sensitivity to undergo degranulation by specific allergens, these methods are not utile for the functional assays of basophils that require longer duration of culture and entire removal of surface IgE.

Monomeric IgE has been shown to support the survival of mast cells in vitro under growth-factor deprived conditions [36]. However, data from the human and mice have shown that IL-3 is critical for the survival of basophils [37]. In general, T cells are the major source of IL-3 [31,32,33,34,35]. The survival of basophils was drastically reduced when cultured with IL-3-deficient T cells. Upon adoptive transfer, T cell-derived IL-3 also induced basophilia in nematode infection model [32]. IL-3 also primes basophil activation and is the major stimuli for the secretion of IL-13 from the basophils [34,38,39,40,41]. Although murine basophils have been shown to be activated by IL-33, TSLP and IL-25 [31], various reports from human show that IL-33, IL-25 and TSLP induce only marginal activation of human basophils [18,42]. Also, TSLP activation of basophils in allergic asthma patients is IL-3 dependent [43]. In line with these reports, we confirm that IL-3 induces the various activation-associated markers in human basophils. Despite culturing the basophils in IL-3, which provides survival and activation signals, acetic acid buffer (pH 4)-treated basophils could not be rescued though cells were exposed to acetic acid buffer (pH 4) only for five minutes on ice. Loss of viability was also observed with acetic acid buffer (pH 6). These data thus provide compelling evidence that acetic acid buffer method of surface IgE stripping is not suitable for the functional assays of basophils. Although cells could be used for the immediate experiments like imaging, sensitization and binding assays, viability would be lost if we culture them.

In line with our current data, previous report has revealed that following exposure of basophils to acetic acid buffer pH 3.7 to elute surface bound IgE, lost the ability to undergo passive sensitization [23]. Even, lactic acid buffer caused damage to the cells and caused lysis of basophils [29]. Although, lactic acid buffer treatment was reduced to 30 s to limit the harm to basophils, the short-term exposure could only remove a small fraction of the cell surface bound IgE [28,29]. For the sensitization assays of basophils, it requires removal of only a small quantity of the IgE from the basophil surface and hence lactic acid treatment for 30 s would serve the purpose. However, partial removal of IgE from the surface of basophils is not sufficient for the functional analyses of anti-IgE IgG autoantibodies. Unlike allergen-specific IgE, these autoantibodies trigger activation of basophils irrespective of antigen specificity [14,19,21]. Therefore, for the functional characterization of anti-IgE IgG autoantibodies it necessitates entire removal of basophil surface-bound IgE. It is not known whether deleterious effects on the basophils are due to acetic acid or pH. As acetic acid buffer (pH 6) was less deleterious to the cells, both pH and acid might have affected viability of basophils.

In view of these shortcomings with acid-based stripping of IgE, we believe that this method could not be used for deciphering the functions of anti-IgE autoantibodies that are found in the healthy individuals and in several pathologies including atopic and non-atopic asthma, chronic spontaneous urticaria, atopic dermatitis and others [13,14,15,18,44,45,46,47,48,49,50]. Previous reports have used myeloma IgE-coupled cyanogen bromide (CNBr)–activated Sepharose 4B columns to deplete anti-IgE autoantibodies from the serum of the patients or from the therapeutic normal IgG [14,18,19]. In addition, micro-well coating of human myeloma IgE protein followed by incubation with purified IgG to remove anti-IgE reactivity has also been attempted [47]. As stripping of IgE on the surface of basophils would lead to an equivalent of ‘IgE knock-out’ basophil model, we anticipated such IgE-deficient basophils would provide convincing evidence that anti-IgE IgG autoantibodies induce human basophil activation. However, in view of adverse effects on the viability and yield, anti-IgE IgG autoantibody depletion strategy appears to be gold standard at present. Another aspect to consider is to reduce the cost of using primary basophils for these experiments. Although may not represent primary basophils from all the aspects, various leukemia cell lines like RBL-2H3 and KU812 are available [51,52,53] and could be explored for testing anti-IgE IgG autoantibodies.

## 4. Materials and Methods

### 4.1. Isolation of Basophils from the Blood

Basophils were isolated from the buffy coats of healthy donors by using Basophil Isolation Kit II (Miltenyi Biotec, Paris, France) and autoMACS (Miltenyi Biotec) [14]. Buffy bags were purchased from Centre Necker-Cabanel, l’Etablissement Francais du Sang (EFS), Paris (INSERM-EFS ethical permission no. 15/EFS/012 dated 22.05.2015) and 18/EFS/033 dated 16.08.2018). The purity of the basophils and the viability as determined by the expression of FcεRI and fixable viable dye (FVD-eFlour506, eBioscience, Paris, France) respectively were over 98%.

### 4.2. Acid Stripping of IgE and Culture of Basophils

Basophils were incubated on ice for 5 min in phosphate buffered saline (PBS) (control) or in ice-cold acetic acid buffer pH 4 (0.05 M acetate, 0.085 M NaCl, 0.01 M EDTA and 0.03% human serum albumin (HSA)) or for 0.5 min in ice-cold lactic acid buffer pH 3.9 (0.13 M NaCI, 0.005 M KCI, 0.01 M lactic acid and 0.03%) [22,23,24,25,26,27,28,54]. Cells were then washed with PBS + 5% HSA and proceeded with phenotype analyses by flow cytometry and/or cultured in serum-free X-VIVO 15 medium (0.1 × 10^6^ cells/well per 200 µL) in 96-well U-bottomed plate along with IL-3 (100 ng/0.5 million cells) for 24 h.

### 4.3. Binding of Exogenous IgE to Basophils

Basophils were incubated with recombinant human IgE (10 ng or 50 ng/million basophils; clone AbD18705_hIgE, AbD Serotec, Bio-Rad, Marnes-la-Coquette, France) for 30 min at 4 °C. Following washing, cells were stained with fluorochrome-conjugated anti-IgE antibodies for the analyses by flow cytometry.

### 4.4. Analyses of Phenotype of Basophils

Basophils were analyzed for the expression of FcεRIa (FcεRIa-FITC, clone CRA-1, Miltenyi Biotec, Paris, France), CD69 (CD69-APC/Cy7, Clone FN50, BD Biosciences, Le Pont de Claix, France), CD123 (CD123-BV421, clone 9F5, BD Biosciences), ST2 (ST2/IL-33R-PE polyclonal goat IgG, R&D Systems, Lille, France) and surface intensity of IgE (anti-IgE–APC, clone MB10-5C4, Miltenyi Biotec) by using LSR II flow cytometer (BD Biosciences) and data were analyzed by BD FACSDiva v8.0.1 (BD Biosciences) and FlowJo v10 softwares (FlowJo, LLC, Ashland, USA). The viability of basophils following isolation was determined by FVD staining. Following culture of basophils for 24 h, viability was analyzed by staining with Annexin V (Annexin V-APC, BD Biosciences) and propidium iodide (PI, InvitrogenTM, Fisher Scientific—France, Illkirch, France).

### 4.5. Statistical Analyses

Prism 6 GraphPad Software (GraphPad Software, San Diego, CA, USA) was used for the statistical analyses. Two-tailed Students’ *t*-test or one-way ANOVA with Tukey’s multiple comparison test were used for the determination of statistical significance as indicated in the figure legends.

## 5. Conclusions

Acetic acid buffer (pH 4) efficiently removes FcɛRI-bound IgE on the basophils. Acetic acid buffer (pH 4)-elution of IgE had no immediate repercussion on the viability of basophils but following 24 h culture with IL-3, the yield and viability of basophils was drastically reduced and had repercussion on the induction of activation markers. Lactic acid treatment on the other hand though had no adverse effects on the viability of basophils and IL-3 induced activation, it removed only a small fraction of the cell surface bound IgE. Thus, acid treatments could be used for the elution of FcɛRI-bound IgE on the basophil surface for the biochemical characterization of IgE antibodies or for the immediate use of basophils to determine their sensitivity to undergo degranulation by specific allergens. However, these methods are not utile for the functional assays of basophils that require longer duration of culture and entire removal of surface IgE.

## Abbreviations

BAFF	B cell activating factor
CNBr	Cyanogen bromide
FcεRI	Type I high affinity immunoglobulin E receptor
FVD	Fixable viable dye
HSA	Human Serum Albumin
IgE	Immunoglobulin E
IgG	Immunoglobulin G
IL	Interleukin
IVIG	Intravenous immunoglobulin
PBS	Phosphate buffered saline
PI	Propidium iodide
TSLP	Thymic stromal lymphopoietin
